# Dynamics and drivers of the human breast milk microbiome across lactation stages

**DOI:** 10.3389/fnut.2026.1861294

**Published:** 2026-07-07

**Authors:** YuQi Xie, YiLing Lu, ZhongHuan Jiang, WeiJie Dong, YiFei Liu, YiFei Wang, Juan Shao, WenLi Yan, ZhiWei Chai

**Affiliations:** 1 School of Food Science and Technology, Shihezi University, Xinjiang, China; 2 The Third Affiliated Hospital of Shihezi University Medical College, Xinjiang, China

**Keywords:** delivery mode, human breast milk, lactation, maternal factors, microbiome

## Abstract

**Introduction:**

Human breast milk (HBM) not only provides all the nutrients and microbiota for the initial development of infants but is also the source of gut microbes in infants and is essential for establishing a healthy gut microbiota and immune system. In this study, we enrolled 188 healthy Uyghur mothers from Xinjiang, China, analysed the structure and diversity of the human breast milk microbial community from 0 to 856 days after delivery, and explored the characteristics of the human breast milk microbiome and potential influencing factors at each lactation stage.

**Methods:**

By constructing microbial cooccurrence relationship networks at various stages of lactation and random forest models, we adopted large sample collection and combined multiple models to largely avoid individual differences.

**Results:**

We showed that the human breast milk microbiota is a dynamic and complex ecosystem, and we found the network central nodes that change during the transition from human colostrum to human mature milk and various stage specific microbial markers. In addition, we also found that the mode of delivery and intrapartum antibiotic prophylaxis during delivery affected the structure and diversity of the microbial community of colostrum, but the degree of influence decreased as the human colostrum transitioned to mature milk.

**Discussion:**

We determined the change trends for microbial community structure and diversity during the transition from human colostrum to human mature milk, provided evidence for the temporal dynamics of the microbial community in human breast milk, and evaluated the impact of potential factors, such as mode of delivery on the microbes in human breast milk. These findings have potential implications for the health and development of infants.

## 1 Introduction

Human breast milk (HBM) is the most ideal source of nutrition for new borns, but its composition varies according to the timing of lactation. Human colostrum (HC) is very rich in essential nutrients (proteins, oligosaccharides, vitamins and minerals), HC is rich in a variety of immunologically active substances. Compared with human mature milk (HM), HC contains higher levels of bioactive factors such as hormones, cytokines, leukocytes, immunoglobulins, lactoferrin, lysozyme, stem cells, human breast milk oligosaccharides (HMOs), microbiota, and microRNAs (1–3). As a result, breast feeding can impact infant health from infancy, through adolescence, and into adulthood. From protecting the infant from infections, to reducing the risk of obesity, type 1 diabetes and childhood leukaemia, many positive health outcomes are observed in infants receiving HBM. For the mother, breast feeding protects against postpartum bleeding and depression, increases weight loss,and longterm lowers the risk of type 2 diabetes, breast and ovarian cancer, and cardiovascular diseases (4–8). From 5 days to 2 weeks postpartum, the TM occurs. One month after childbirth, the HBM achieves a standard composition, known as ‘HM’. In contrast to HC, HM is characterized by a lower concentration of proteins and more abundant of lipids and carbohydrates, such as lactose and oligosaccharides (8, 9), indicating that they may support the nutritional and developmental needs of growing infants more significantly. Therefore, the World Health Organization (WHO) and the United Nations International Childrens Emergency Fund (UNICEF) recommend that children initiate breast feeding within the first hour of birth and be exclusively breastfed for 6 months (10). Interestingly, as the infant matures, the concentrations of many HBM immune compounds decline during lactation (from HC to HM), suggesting that certain functions of HBM would be specifically potentiated or weaken at different stages of lactation (11–14).

Notably, research in the past few decades has revealed the diversity of the microbial community in HBM. The main bacteria species include *Staphylococcus*, *Streptococcus*, *Lactobacillus*, and *Bifidobacterium* (15–17). Diverse microorganisms present in the initial streams of HBM modulate the colonization of microbiota in the small intestine of infants and it has been recognized that it significantly affects the development and maturation of the infant’s immune system. Thus, breast feeding is considered critical for the overall growth and development of newborns as it establishes local and systemicimmune tolerance to the foreign molecules ingested during breast feeding (18, 19). Leading to an increasing interest in HBM microbiota and their effects in maternal infant health. It is widely believed that HBM provide a commensal ‘inoculum’ for their neonates or infants gastrointestinal development, establishing a healthy intestinal microbiome (20–22).

Multiple studies have documented that the richness and diversity of HBM microbiota vary greatly within and across cohorts (16, 23–25). It is worth noting that the mother’s ethnicity, physical health, and mode of delivery can all have an impact on the infant’s gut microbiota (26–28, 54). Meanwhile, other studies found that HBM over the course of lactation (HC, TM and HM) showed a differentiable pattern of bacterial diversity and structure composition, and the bacterial diversity of HC was higher than that of HM and TM (16, 22, 29). However, reports on the differences of HBM microbiome across different lactation periods are still fewer. The conclusions are even contradictory. Furthermore, a greater abundance of anaerobic intestinal bacteria in HM has been observed compared with HC samples (30, 31). For example, *Bifidobacterium.* were sporadically recovered in culture media or found in a small portion of HC samples tested, and their relative proportion and prevalence is very low. More recently, the prevalence of bifidobacteria in transitional and HM has been reported to be increased to some extent (32–34). Overall, there is no consensus on whether the diversity and structure of the HBM microbiome in cohort of healthy individuals from different ethnicity varies regularly with lactation time. One important reason is the small cohort studied, and another is that there are many factors that are intertwined with the HBM microbiome.

In this study, we recruited 188 healthy mothers who live in a limited rural area of Kashgar, western China’s Xinjiang, where relatively closed cultural traditions are maintained, and lifestyles and eating habits are unique. In particular, because of ethnic habits and customs, a large proportion of mothers have a long lactation period, even longer than 24 months. According to the specificity of the cohort, this study will provide strong data to elucidate the temporal variability of the HBM microbiome of a cohort of mothers residing in a narrow rural area and potential influencing factors.

## 2 Materials and methods

### 2.1 Sample collection

We collected HBM from mothers between 1 and 856 days after birth, during clinic or home study visits and recruited mother-infant pairs meeting the following criteria: (i) the Uyghur people native to Kashgar, Xingjiang, (ii) either vaginal delivery at full-term (≥37 weeks gestation) or caesarean section at full-term (≥37 weeks gestation), (iii) exclusive breast feeding during the first 5 months and the lactation continuing until sampling. For mothers who underwent caesarean section, intrapartum antibiotic prophylaxis (IAP) was administered according to standard clinical practice. All participants (except for the routine IAP in the caesarean section group) reported no use of systemic antibiotics or probiotic supplements within 1 week prior to sampling. The inclusion criteria were identical for both delivery mode groups, except for the delivery mode itself, to ensure comparability between groups. All the participants were healthy and do not require hospitalization. They were included for microbiota analysis with standard collection protocol (5, 35). The study was conducted according to the guidelines in the Declaration of Helsinki. The Human Research Ethics Boards at Shihezi University provided institutional review board approval (Reference number: KJ2022-079-01). Written informed consent was obtained from all subjects participating in the study.

Demographic and clinical data were recorded in a specific case report form. All participants responded to a general questionnaire including socio-economic, lifestyle aspects and body mass index (BMI) of the mother. Maternal weight was measured to the nearest 0.1 kg using a calibrated digital scale (light clothing, no shoes), and height was measured to the nearest 0.1 cm using a wall-mounted stadiometer. BMI was calculated as weight (kg)/height^2^ (m^2^). The case report recorded the number of gestational weeks at delivery, delivery method, feeding patterns, and IAP.

Standard sterile collection tubes were using to collect and HBM (with the aid of a breast pump) samples, and the first few drops (0.5–1 mL) were discarded, and the breast was thoroughly cleansed with chlorhexidine solution before manually collecting 3–5 mL of milk. Samples were immediately transported to the laboratory using portable refrigerators and ice packs. Each HBM sample were divided into several 1 mL servings into sterile centrifuge tube ready for DNA extraction and then were all frozen at −80 °C in batches for processing and remained frozen until DNA extraction. Lactation stages were defined as follows: colostrum (0–6 days), transitional milk (6–15 days), and mature milk (≥16 days). Days of lactation (DAY) was treated as a continuous variable in all multivariate models to account for the wide range of sampling times in the mature milk group (16–856 days). Total bacterial DNA from all samples was extracted within 7 days of sampling and sequenced to minimize errors due to storage, experimental conditions, and sequencing.

### 2.2 DNA extraction and high-throughput sequencing

For HBM samples, a TIANamp Blood DNA Kit (TIANGEN, Beijing, China) was utilized to extract bacterial DNA with some modifications to a previously reported protocol (5, 35). One millilitre of sample was centrifuged at full speed (13,000xg) for 15 min at 4 °C. The fat rim was carefully removed using a sterile swab, and the pellets were resuspended in 200 μL of Tris-EDTA (TE) buffer and treated with 10 μL of lysozyme (50 mg/mL) and 5 μL I of DNase-free RNase (20 mg/mL) for 30 min at 37 °C. Then, 25 mg of glass beads (50 μm) were added to the solution and treated with 3 beadbeating steps in a FastPrep instrument (MP Biomedicals, Irvine, CA, United States) at 5.5 movements per sec for 1 min. After pulse centrifugation, 20 μL protease K and 200 μL of GB buffer was added to the supernatants. Followed by a water bath at 56 °C for 30 min, 200 μL of ethanol were add to the supernatants. And total DNA was further purified from the supernatant using Spin Columns CB3 (TIANamp Blood DNA Kit) following the manufacturer’s instructions.

### 2.3 Sequencing data processing

The total DNA in each HBM sample was subjected to bacterial 16 s rRNA V4-V5 amplification and high-throughput sequencing by Shanghai Majorbio Bio-Pham TechnologyCo Ltd.^1^ The paired-end sequencing data were imported into QIIME2, the barcode was removed through the cutadapt module, and then, the dada2 module was used to sequentially perform quality control, remove paired-end primers, and remove redundancy and noise, obtaining a feature table and the representative sequence of each ASV. The V4-V5 section of the Greengenes reference database was trained through the fit-classifier-naive-bayes classifier to generate a classification set specific to this study. Each feature sequence was compared and annotated with the classification set with 99% sequence similarity. Samples with less than 10,000 reads (*n* = 2) and ASVs with less than 5 occurrences in all samples were excluded, leaving a total of 867 unique ASVs for subsequent analysis. The FastTree method was used to construct a phylogenetic tree. The phenotypic characteristics of microorganisms were identified and counted through BugBase.^2^ The observed ASVs and Shannon indexes were calculated by QIIME 2 to represent alpha diversity, representing richness and diversity, respectively. Bray-Curtis distance matrix and weighted and unweighted UniFrac distances were calculated by QIIME 2 to represent the beta diversity.

### 2.4 Microbiome profiling and statistical analysis

No formal sample size calculation was performed prior to the study, as this was an exploratory investigation aimed at characterizing the breast milk microbiome in a previously unstudied population (Kashgar Uyghur mothers). The final sample size (*n* = 188) was determined by the number of eligible mother-infant pairs who consented to participate during the recruitment period. This sample size is comparable to or larger than similar studies on human milk microbiota. All statistical analyzes and data visualization were performed in R (version 4.4.3) (36). Linear regression was used to evaluate the association of maternal factors with the relative abundance of *Actinomycetota* and alpha diversity, adjusting for factors with a *p* value < 0.05 in univariate analysis using the cor.test function in psych package and visualization was performed by ggplot2. In the analysis of correlation test, Pearson correlation coefficient was used for continuous variables (maternal age, BMI, parity and days of lactation) and Spearman was for ordinal variables (stage of lactation, level of maternal age). A *t*-test was used to assess the dissimilarity of alpha diversity between different modes of delivery. The Bray-Curtis distance matrix was calculated to identify clusters of the HBM microbiome by the hierarchical clustering method using factoxetra package. Principal coordinates analysis (PCoA) on weighted and unweighted UniFrac distances was performed using the cmdscale function in vegan package and plotted the samples by the first two coordinates. The contribution and dissimilarity of clusters and lactation on beta diversity were tested by adonis function using R package vegan with 10,000 permutations (37). We considered *p* < 0.05 to be significant. All *p*-values were corrected by the false discovery rate (FDR) using the Benjamini–Hochberg (38) method for multiple comparisons. Linear discriminant analysis (LDA) effect size (LEfSe) was used to assess taxon enrichment based on clusters and level of maternal age with default parameters (39) and a logarithmic LDA score threshold of two using open reference strategy.^3^ Co-occurrence relationship of HBM microbiota at various stages of lactation was determined based on the Spearman correlation coefficient and was visualizated using Annotation DBi package. The lactation days and stages discriminatory bacterial taxa were identified by applying random forest regression of their relative abundance in HBM using random Forest package.

## 3 Results

### 3.1 Microbial community structures in breast milk

**Figure 1:**
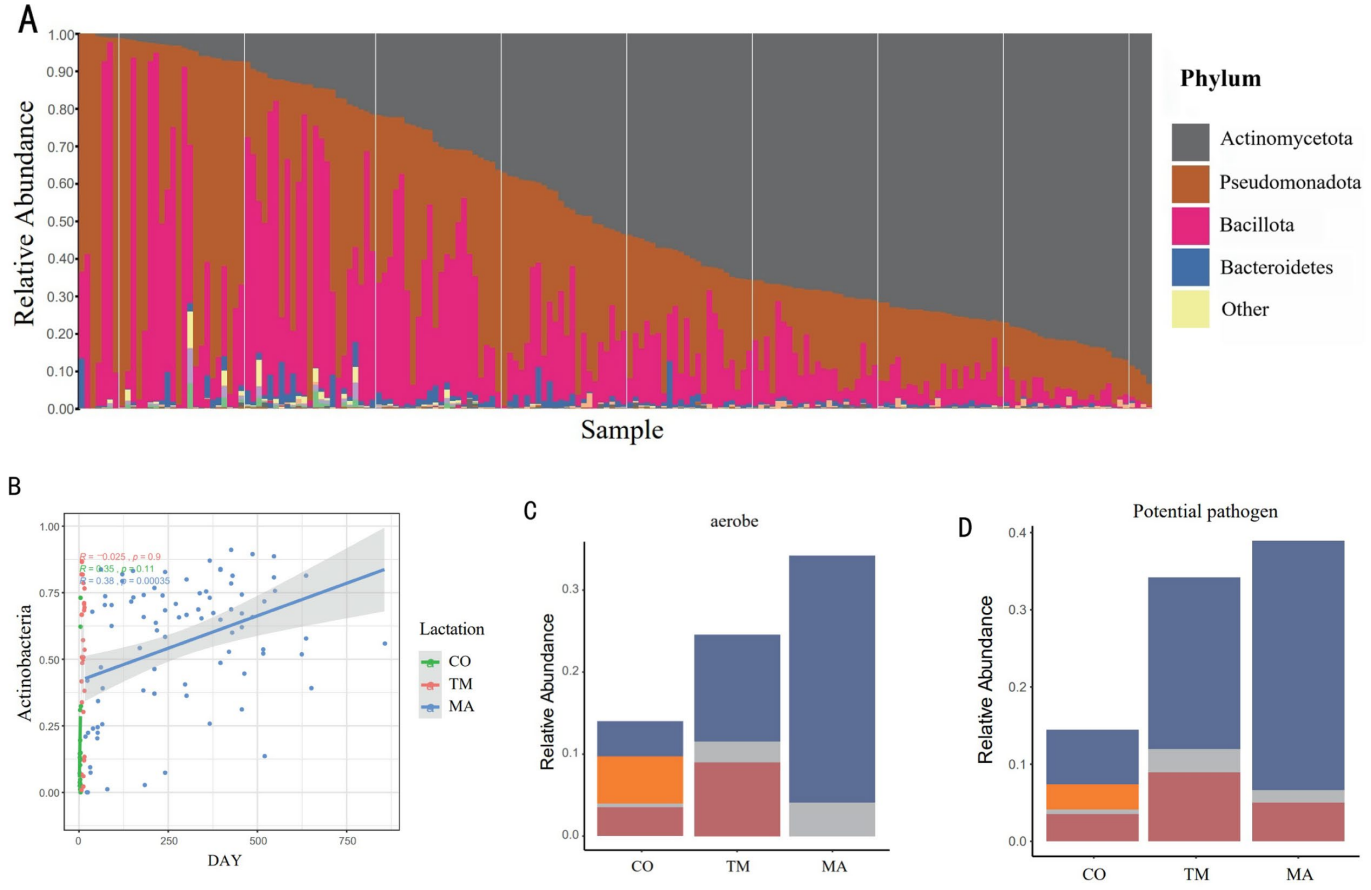
**(A)** Relative abundance of dominant bacterial phyla in HBM samples. **(B)** Relationship between days postpartum and the relative abundance of *Actinomycetota* in HBM. **(C)** Relationship between aerobic microorganisms and milk maturation. **(D)** Relative abundance of pathogens in HC.

Among microbiota in all HBM samples in this study, the 3 most dominant phyla were Actinomycetota (average relative abundance, 57.4%), Pseudomonadota (average relative abundance, 26.4%) and Bacillota (average relative abundance, 14.2%) (Figure 1A).

As the common human skin bacteria, *Micrococcaceae* was the most dominant family of HBM in our study and its average relative abundance (54.9%) accounted for 95.6% of the relative abundance of *Actinomycetota*. However, the most dominant member belong to *Micrococcaceae* at the genus level were those that have not been classified into known genera (unassigned *Micrococcaceae*) but identified as aerobe according to the result of BugBase analysis. *Enterobacteriaceae* was the most dominant family for *Pseudomonadota* with an average relative abundance of 12.8%; and among its members at genus level, *Serratia* was the most prominent one. The average relative abundance of Serratia was 7.8% which was second only to that of unassigned *Micrococcaceae* of *Actinomycetota*. For *Bacillota,* the predominant families were *Streptococcaceae* (average relative abundance, 6.3%; average relative abundance of *Streptococcus*, 5.7%) and *Staphylococcaceae* (average relative abundance, 3.5%; average relative abundance of *Staphylococcus*, 3.4%). In addition, the relative abundance of *Acinetobacter* and *Citrobacter*, which belong to the family *Moraxellaceae* and *Enterobacteriaceae* were remarkable higher (3.1 and 2.7%, respectively) than other genera.

However, there were large individual differences in the microbial community structure of HBM, and these differences were mainly manifested in the proportions of *Actinomycetota* to *Pseudomonadota* and *Bacillota*. In most samples, *Actinomycetota* was negatively correlated with *Pseudomonadota* and *Bacillota*, and according to the variance test results (Tables 1, 2), the proportions of these 3 phyla were significantly correlated with mode of delivery (*p* = 0.001), IAP (*p* = 0.01) and number of days of lactation (DAY) (*p* = 0.001), while having no correlation with maternal age and BMI (*p* > 0.05). For all mothers who had a natural delivery and were beyond 15 days of lactation, DAY was proportional to the relative abundance of Actinomycetota in HBM (Figure 1B) and inversely proportional to the relative abundance of *Pseudomonadota* and *Bacillota*.

**Table 1:** Relevant factors affecting the abundances of *Actinomycetota, Pseudomonadota*, and *Bacillota* in HBM samples.

Shannon	observed_ASVs					
	Df	Sums of squares	Mean squares	F.Model	Variation (*R*^2^)	Pr (>F)
DEL	1	0.265511564	0.265511564	6.986419928	0.040974486	0.00669933
ANT	1	0.033819677	0.033819677	0.889898961	0.005219147	0.358664134
AGE	1	0.039256356	0.039256356	1.032954574	0.00605815	0.327267273
BMI	1	0.047559663	0.047559663	1.251439954	0.00733954	0.266173383
BMI_L	2	0.054781081	0.027390541	0.720728755	0.008453969	0.508449155
AGE_L	2	0.091205426	0.045602713	1.199946621	0.014075076	0.304269573
NUM	1	0.018363094	0.018363094	0.483189083	0.002833844	0.538446155
ROW	2	0.062758207	0.031379104	0.825680023	0.009685022	0.447955204
DAY	1	0.036115503	0.036115503	0.950309146	0.005573445	0.341865813
cluster	3	0.16796493	0.05598831	1.47322338	0.025920817	0.204979502

**Table 2:** Maternal factors vs. alpha diversity of human milk microbial communities.

Shannon	Observed_ASVs						
	Df	Sums of squares	Mean squares	F.Model	*R* ^2^	Pr (>F)	fdr
DEL	1	0.265511564	0.265511564	6.986419928	0.040974486	0.00669933	DEL
ANT	1	0.033819677	0.033819677	0.889898961	0.005219147	0.358664134	ANT
AGE	1	0.039256356	0.039256356	1.032954574	0.00605815	0.327267273	AGE
BMI	1	0.047559663	0.047559663	1.251439954	0.00733954	0.266173383	BMI
BMI_L	2	0.054781081	0.027390541	0.720728755	0.008453969	0.508449155	BMI_L
AGE_L	2	0.091205426	0.045602713	1.199946621	0.014075076	0.304269573	AGE_L
NUM	1	0.018363094	0.018363094	0.483189083	0.002833844	0.538446155	NUM
ROW	2	0.062758207	0.031379104	0.825680023	0.009685022	0.447955204	ROW
DAY	1	0.036115503	0.036115503	0.950309146	0.005573445	0.341865813	DAY
cluster	3	0.16796493	0.05598831	1.47322338	0.025920817	0.204979502	cluster

For mothers who had a natural delivery and less than 15 days of lactation and for all the mothers who delivered by caesarean section, the relative abundance of these 3 phyla was not significantly correlated with DAY (*p* > 0.05).

We used BugBase to identify the phenotypic characteristics of HBM microbiota. We found that the relative abundance of aerobic microorganisms increased with the increase in HBM maturity (Figure 1C).

Among them, most of the aerobic microorganisms in HC samples belonged to *Bacillota* (62%), most of the aerobic microorganisms in TM samples belonged to *Pseudomonadota* (51%) and *Actinomycetota* (38%), and most of the aerobic microorganisms in HM samples belonged to *Pseudomonadota* (87%); no aerobic microorganisms under the phyla *Bacillota* and *Actinomycetota* were identified. In addition, the relative abundance of potential pathogens in HC (14%) (especially *Bacillota*) was also much lower than that in TM (34%) and HM (39%) (Figure 1D). Regardless of whether the ASVs were identified *as* gram-negative or as gram-positive, their relative abundances in HC (23% negative, 7% positive) were much lower than those in TM (38% negative, 17% positive) and HM (45% negative, 15% positive), indicating that HC contained more species that could not be identified.

### 3.2 Breast milk microbial diversity in each lactation stage

**Figure 2:**
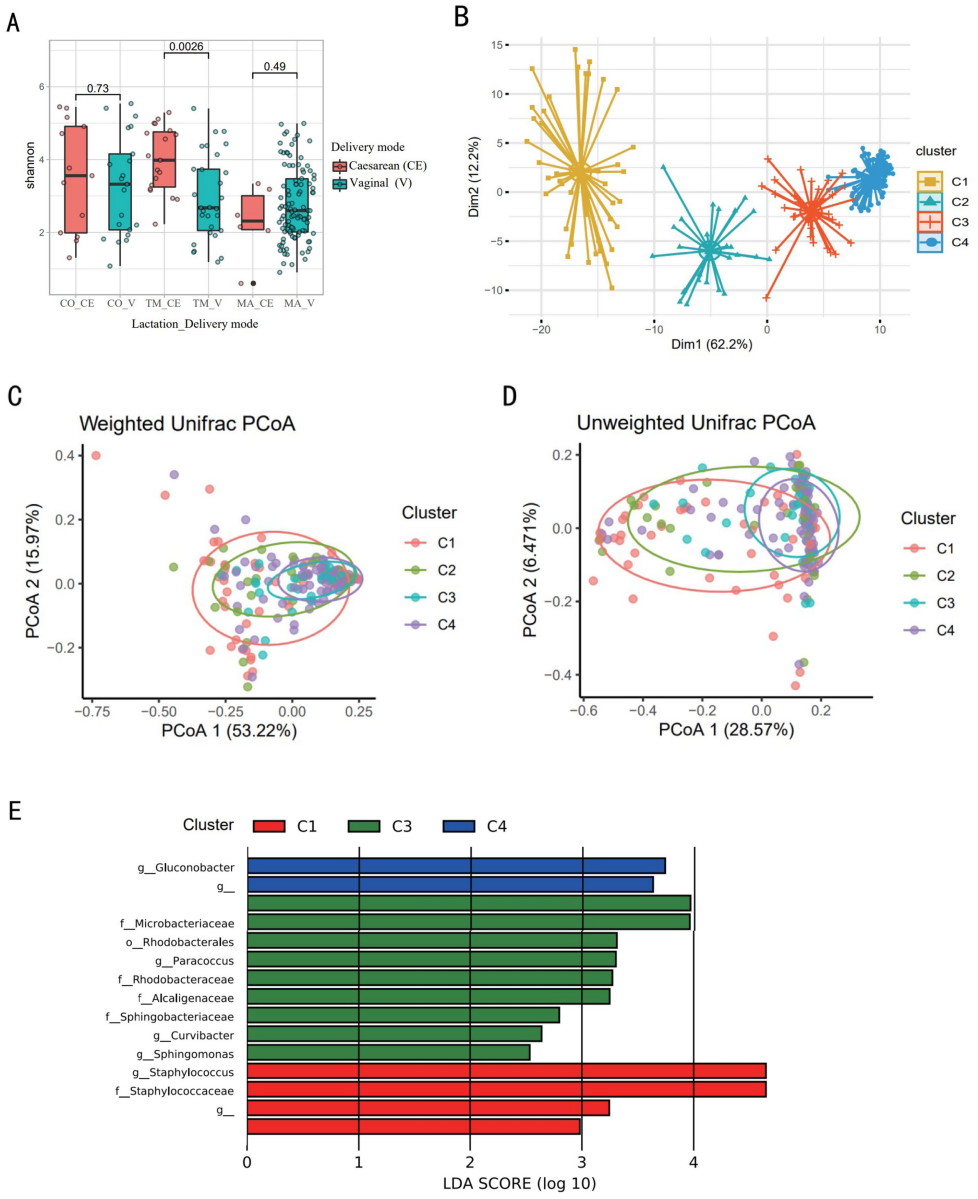
**(A)** The influence of delivery methods on different lactation periods. **(B)** Hierarchical clustering analysis of HBM samples based on Bray-Curtis distance resulted in four distinct clusters. **(C,D)** The distances of weighted and unweighted UniFrac. **(E)** LEfSe analysis of the relative abundance of microorganisms in the four clusters.

To explore the diversity of HBM microbial communities at various stages of lactation, we analysed alpha and beta diversity. We calculated the observed amplicon sequence variants (ASVs) and Shannon index in each HBM sample to represent alpha richness and diversity, respectively. The result of univariate analysis of variance (ANOVA) indicated that the richness (mean ± SD = 90.49 ± 34.87) and diversity (3.02 ± 1.17) of HBM were not significantly correlated with DAY, IAP, maternal age and BMI (*p* > 0.05), but that diversity was significantly correlated with mode of delivery (*p* = 0.04) (Table 3). We also found that mode of delivery had a significant impact only on TM (TM, within 6–15 days of lactation) (*p* = 0.003); mode of delivery had no significant impact on HC (CO) and HM (MA) (*p* > 0.05) (Figure 2A).

**Table 3:** Related influencing factors of HBM richness.

		Df	Sums of squares	Mean squares	F.Model	Variation (*R*^2^)	Pr (>F)
ROW	Group	2	3.977810969	1.988905485	12.30333233	0.117957232	1.00E-04
	Residuals	184	29.74467400	0.161655837		0.882042768	
	Total	186	33.72248497			1	
DEL	Group	1	0.842928661	0.842928661	4.596156794	0.026476144	0.00049995
	Residuals	169	30.99436118	0.183398587		0.973523856	
	Total	170	31.83728984			1	
DAY	Group	1	2.560779455	2.560779455	15.20276863	0.075936855	1.00E-04
	Residuals	185	31.16170552	0.168441651		0.924063145	
	Total	186	33.72248497			1	
BMI	Group	1	0.168994909	0.168994909	0.931767696	0.005011342	0.446755324
	Residuals	185	33.55349006	0.181370217		0.994988658	
	Total	186	33.72248497			1	
BMI_L	Group	2	0.322088781	0.161044391	0.887180131	0.009551158	0.578942106
	Residuals	184	33.40039619	0.181523892		0.990448842	
	Total	186	33.72248497			1	
ANT	Group	1	1.027637046	1.027637046	5.572936936	0.0330595	1.00E-04
	Residuals	163	30.05683365	0.184397752		0.9669405	
	Total	164	31.0844707			1	
AGE	Group	1	0.512360404	0.512360404	2.854149932	0.015193436	0.005799
	Residuals	185	33.21012457	0.179514187		0.984806564	
	Total	186	33.72248497			1	
AGE_L	Group	2	1.04845696	0.52422848	2.952131898	0.031090738	0.0013
	Residuals	184	32.67402801	0.177576239		0.968909262	
	Total	186	33.72248497			1	

Furthermore, at the various lactation stages, there was no significant difference in the microbial alpha diversity in HBM from mothers who had a natural delivery (*p* > 0.05) while microbial alpha diversity of HBM from mothers who underwent caesarean section increased first and then decreased with progression through lactation stages; the microbial diversity in HC from the mothers who underwent caesarean section varied greatly among samples. Additionally, we also found that the microbial alpha diversity in HBM from mothers who had a natural delivery and that in HBM from mothers who underwent caesarean section showed the largest difference between HC samples and the smallest difference between HM samples.

To better understand the causes for the individual differences among HBM samples in the above results, we utilized human gut microbial community typing. We hierarchically clustered HBM samples based on the Bray-Curtis distance and classified all HBM samples, eventually identifying 4 clusters (Figure 2B).

Principal coordinates analysis (PCoA) results show that independent of the weighted (Figure 2C) or unweighted UniFrac distance (Figure 2D), the C3 and C4 groups were densely clustered, with no significant difference between the 2 groups (variance test, *p* > 0.1); the C1 and C2 groups were relatively scattered, with no significant difference between them (variance test, *p* > 0.1). A pairwise comparison of C1 and C2 with C3 and C4 showed significant differences between the groups (*p* < 0.05).

Through LEfSe analysis (Figure 2E), it was found that the average relative abundance of *Staphylococcus* in C1 was 11.60%, but much higher than those in C3 (3.63%) and C4 (4.65%). Although the genus *Sphingomonas* was a biomarker in C3 (LDA effect size = 2.58, *p* = 0.01), its average relative abundance in C3 was less than 0.04%, even less than that in the cluster (0.02%). Unassigned *Enterobacteriaceae* (LDA effect size = 4.06, *p* < 0.001) and *Gluconobacter* (LDA effect size = 4.04, *p* = 0.02) at the genus level were both biomarkers in the C4 cluster; their average relative abundances in C4 were 1.13 and 1.09%, respectively.

**Figure 3:**
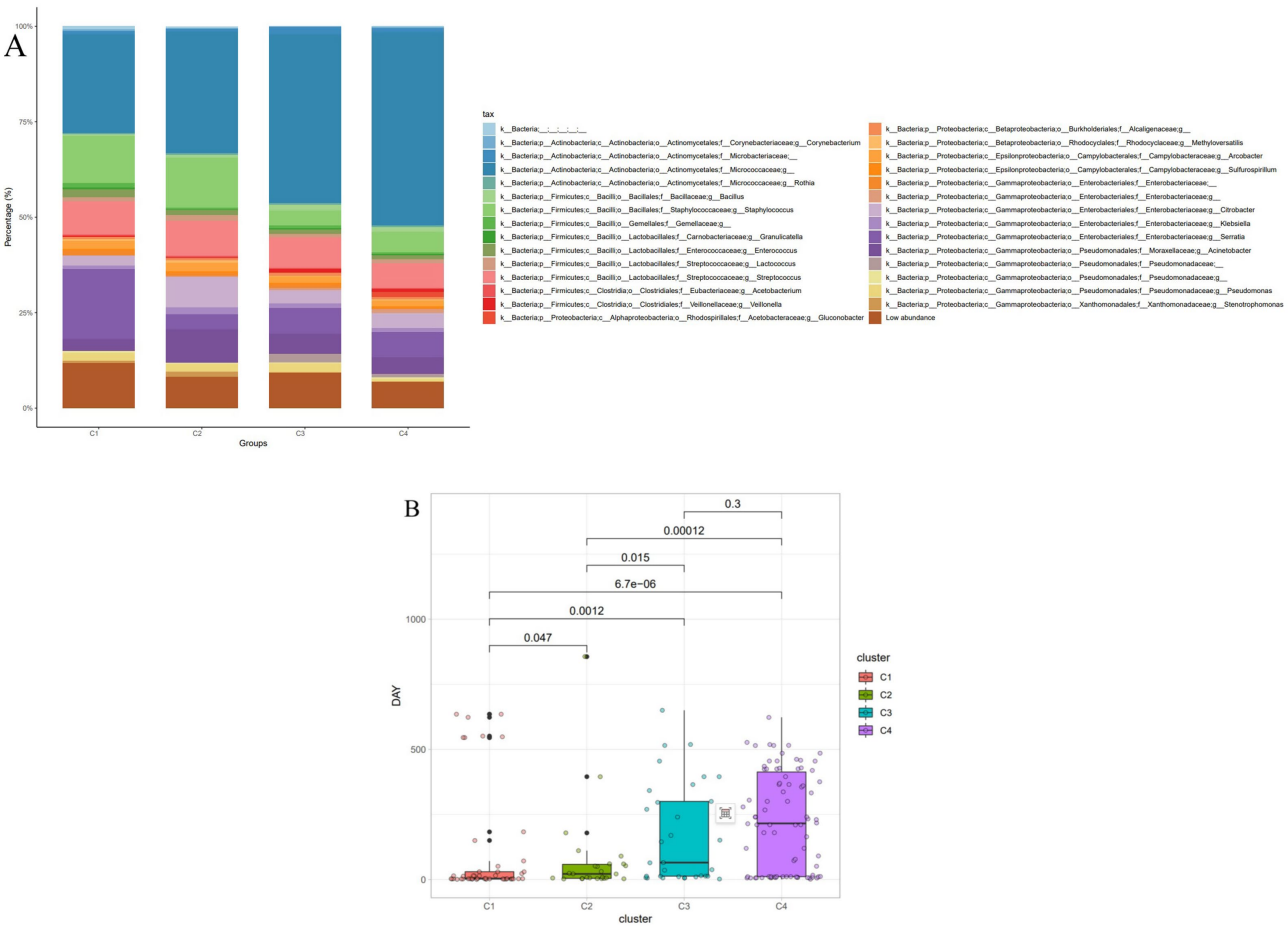
**(A)** Relative abundance variations of different genera across clusters C1-C4. **(B)** Box plot of the distribution of cluster samples by age in days.

In addition, based on the average relative abundances of the 25 most dominant genera in each cluster (Figure 3A), we found that unassigned *Micrococcaceae* showed an increasing trend in groups C1-C4 while *Staphylococcus*, *Serratia*, *Enterococcus*, and *Lactococcus* showed decreasing trends. Through the chi-square test, we found that the microbial community type (cluster) in HBM was significantly correlated with DAY (*p* < 0.001) only. The mean ± standard deviation (SD) days for C1, C2, C3, and C4 cluster HBM were 104 ± 209, 79 ± 178, 176 ± 191, and 224 ± 189, respectively. It can be seen from the box plot (Figure 3B) that the distribution of samples in clusters C1-C4 on DAY gradually increased, and except for C3 and C4, the differences among the groups were significant (rank sum test, *p* < 0.05). HC samples (less than 6 days of lactation) accounted for a large proportion of C1 (25/46), significantly higher than the proportion of HC samples in clusters C3 and C4 (*p* < 0.001). The proportions of HM samples (more than 15 days of lactation) were 13.3% (14/26), 19.0% (20/33) and 53.3% (56/82) in clusters C2, C3, and C4, respectively showing an increasing trend. These results showed that the characteristic microbial composition and beta diversity of each cluster and its change rule may also be the sign and rule of HBM maturation.

**Figure 4:**
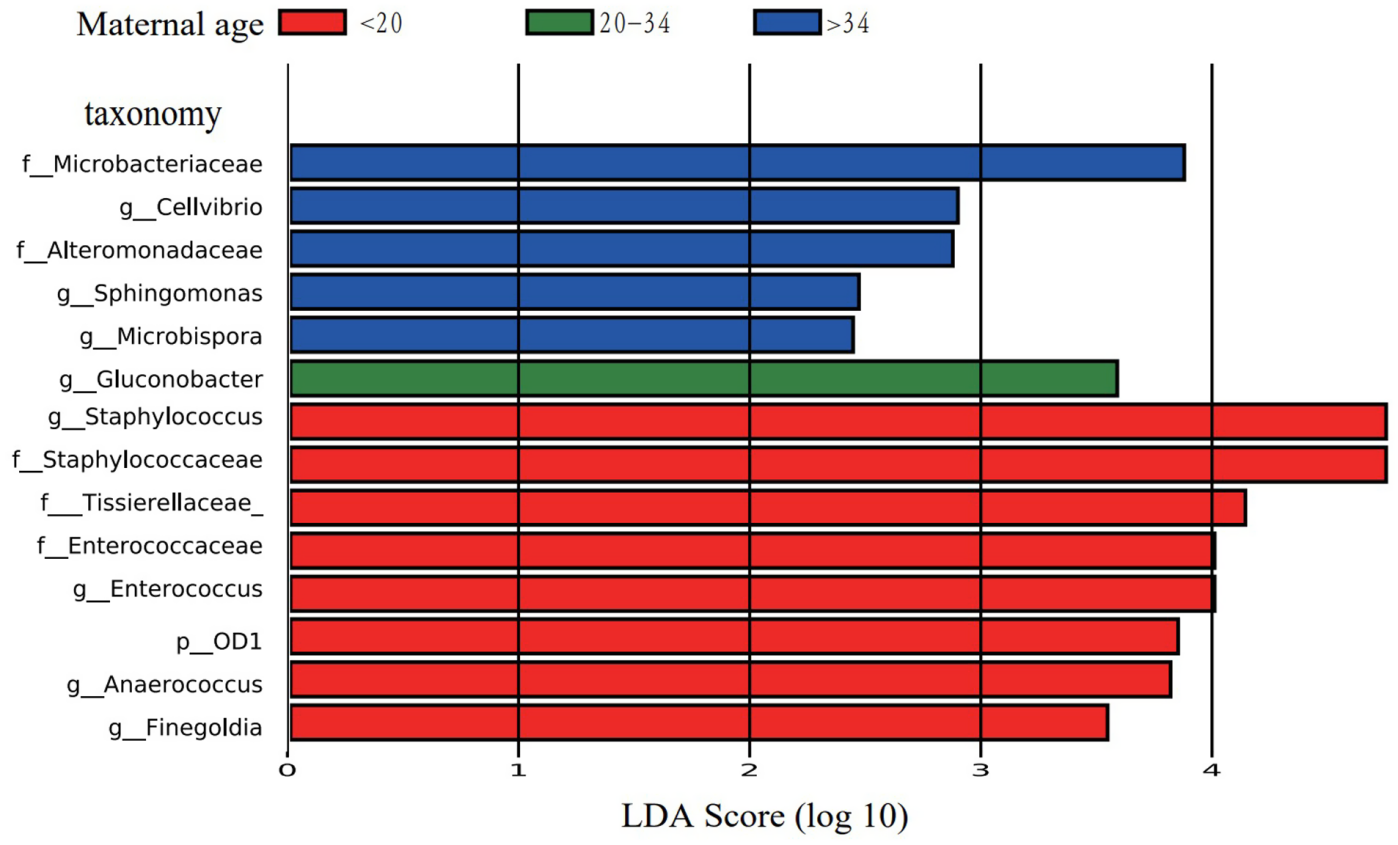
The abundance of HBM microorganisms at different ages.

Next, we calculated the weighted and unweighted UniFrac distances for the beta diversity in HBM. Through ANOVA, we found that DAY, mode of delivery, IAP, parity, and maternal age significantly affected the beta diversity in HBM (*p* < 0.05). Through the chi-square test results, we found that maternal age was only significantly correlated with parity (*p* < 0.001) and not significantly correlated with maturity and other factors (*p* > 0.05). The results of the pairwise analysis of the UniFrac distance for each factor showed that independent of the unweighted or weighted UniFrac distance, there were significant differences among the HBM from primiparous mothers in the young age group (<20 years) and that from multiparous mothers in the middle (20–34 years) and older (>35 years old) age groups (*p* < 0.001). These differences are mainly manifested as follows: the average relative abundance of *Staphylococcus*, *Enterococcus* and *Anaerococcus* (8.4, 3.7, and 1.3%, respectively) in the HBM from primiparous mothers in the young age group was significantly higher than that from mothers in other groups (LDA effect size>3.8, *p* < 0.05) (Table 3). The average relative abundance of unassigned Microbacteriaceae in HBM from multiparous mothers in the older age group (1.5%) was significantly higher than that in HBM from mothers in other groups (LDA effect size>4, *p* < 0.01) (Figure 4).

**Figure 5:**
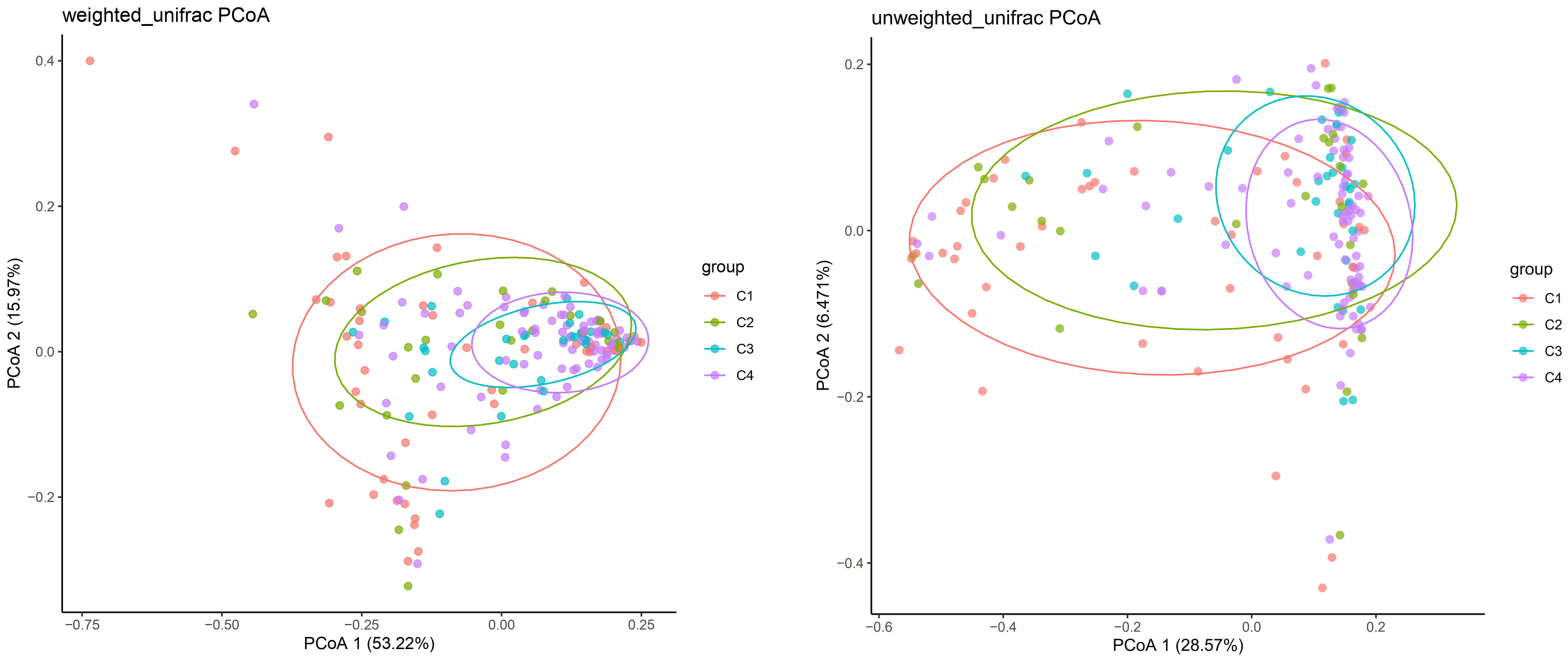
Analysis of weighted and unweighted Unifrac distances.

Mode of delivery was significantly correlated with IAP and HBM maturity (HC, TM and HM) (chi-square test, *p* < 0.001). However, PCoA analysis based on weighted and unweighted Unifrac distance (Figure 5)showed that delivery mode only had significant influence on transition milk (both *p* < 0.001), but no significant influence on both HC (PERMANOVA; *p* = 0.62, *p* = 0.67, respectively) and HM (*p* = 0.29, *p* = 0.52, respectively). Moreover, there was no significant difference of beta diversity (weighted and unweighted Unifrac) between TM and HC in caesarean delivery group (*p* = 0.15, *p* = 0.28), while the difference of unweighted Unifrac diversity between TM and HC in vaginal delivery group and HM was significantly (*p* < 0.01; weighted Unifrac: *p* = 0.25) (see Table 4).

**Table 4:** The abundance of different bacteria in different lactation periods.

CO			CM			MA			
Factors	*R* ^2^	Pr (>F)	fdr	*R* ^2^	Pr (>F)	fdr	*R* ^2^	Pr (>F)	fdr
DEL	0.004660318	0.767	0.876571429	0.169632043	0.003	0.024	0.018536178	0.176	0.372
ANT	0.035785523	0.384	0.673333333	0.001472585	0.877	0.877	0.002800355	0.684	0.699
AGE	0.001480026	0.932	0.932	0.01626462	0.379	0.6992	0.012005068	0.286	0.4576
BMI	0.042411394	0.243	0.673333333	0.015887814	0.411	0.6992	0.092257787	0.002	0.016
BMI_L	0.057083058	0.446	0.673333333	0.014605426	0.718	0.820571429	0.098105313	0.011	0.044
AGE_L	0.015432518	0.505	0.673333333	0.053102786	0.278	0.6992	0.033285997	0.186	0.372
NUM	0.038266195	0.29	0.673333333	0.005266765	0.648	0.820571429	0.002857693	0.699	0.699
DAY	0.03362672	0.299	0.673333333	0.013797352	0.437	0.6992	0.003892634	0.655	0.699

When HBM maturity was controlled (because mature HBM samples from mothers who underwent caesarean section accounted for less than 6% of all samples, HM was excluded, and only HC and TM were considered), we found that mode of delivery and IAP had no significant impact on the beta diversity in HC and TM (*p* > 0.05). Although the delivery mode did not show any effect on the beta diversity of HC microbe, it was noteworthy that the individual differences of beta diversity within-group of HC microbe were still the largest among all groups, especially the HC of caesarean section mothers, while the differences in the HM were relatively small and concentrated in both caesarean section and vaginal delivery groups. These results suggested that the effect of delivery mode and IAP on the microbial beta diversity of HBM depends on the stage of lactation.

Although we determined the degree of influence of these factors on the beta diversity in HBM, due to the large individual differences, DAY, maternal age, BMI, and parity in the redundancy analysis results only explained 10.2% of the structure and diversity of the microbial community in HBM (*R*^2^ = 0.102, *p* < 0.001). When using redundancy analysis to classify various factors, maternal age, BMI, parity, and lactation stage explained 19.4% of the structure and diversity of the microbial community in HBM (*R*^2^ = 0.194, *p* < 0.001), of which lactation stage explained 18.5% (*R*^2^ = 0.185, *p* < 0.001).

### 3.3 Co-occurrence relationship of breast milk microbiota at various stages of lactation

**Figure 6:**
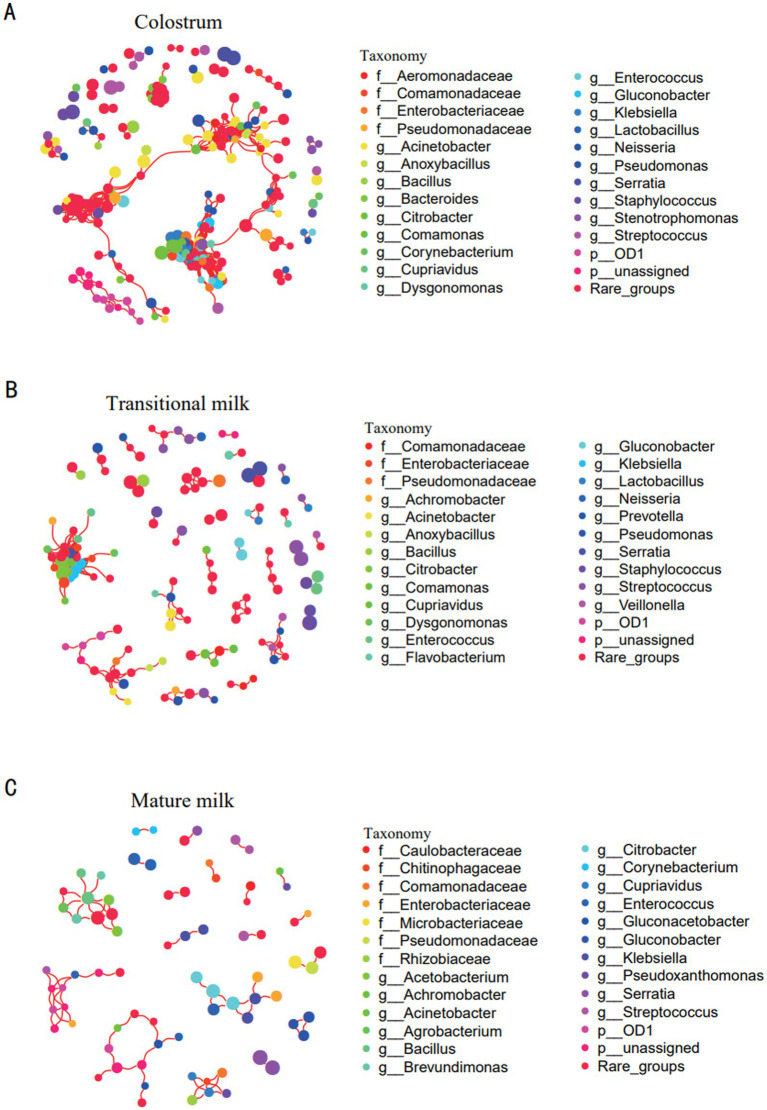
**(A)** HC network topology. **(B)** TM network topology. **(C)** HM network topology.

To explore the co-occurrence relationship of HBM microbial communities at various stages of lactation, we constructed a co-occurrence relationship network diagram based on the Spearman coefficient for the microbial community structure at the level of ASVs. No negatively correlated ASVs were found in the co-occurrence networks for the 3 stages of HBM. The plot shows that the topology of the HC microbial co-occurrence relationship network (Figure 6A) was the most complex (connectance = 0.033), with the highest average degree (average degree = 8) and the most complex ecological cluster (clustering coefficient = 0.63), but the degree of separation was also the highest (average path length = 7.51); the HM network (Figure 6C) topology was the simplest (connectance = 0.028), with the lowest average degree (average degree = 2.08) and the fewest ecological clusters, which were scattered (cluster = 19, clustering coefficient = 0.43); and the network topology for TM (Figure 6B) was between those for HC and HM. In the co-occurrence network of HC microorganisms, 6 of all genera with a degree greater than 15 (*n* = 17) were determined to be aerobic microorganisms, and 8 of all genera with a degree greater than 15 were facultative anaerobic microorganisms.

In this correlation-based co-occurrence network, the nodes with the highest degree (number of connections) were *Serratia* (degree = 28), *Achromobacter* (28), *Gluconacetobacter* (26), *Elizabethkingia* (26), and *Klebsiella* (25). In the HC network, the ASVs with the highest betweenness centrality were *Sphingobacterium faecium* (betweenness = 5,292), unassigned *Microbacteriaceae* (5262) and unassigned *Aeromonadaceae* (5249). In the co-occurrence network of TM microorganisms, there were 2 aerobic microorganisms and 7 facultative anaerobic microorganisms in all genera with a degree greater than 20 (*n* = 10), among which the nodes with the highest degree were *Klebsiella* (26), *Comamonas* (24), *Serratia* (23) and *Enterococcus haemoperoxidus* (22), and the nodes with the highest betweenness centrality were *Klebsiella* (57) *Vogesella* (50) and *Wautersella* (36). In this correlation-based co-occurrence network, the nodes with the highest degree were *Bacillus* (8), *Cupriavidus* (5), *Arcobacter* (5) and *Klebsiella* (4), and the nodes with the highest betweenness centrality were Bacillus (24), Gluconacetobacter (17) and *Enterococcus* (14). We found that *Klebsiella*, which is common in the human intestine, had the highest degree in all three stages, indicating it was highly connected in the correlation network. *Serratia*, another common genus in human intestines, also gradually lost its high degree centrality with the increased maturity of HBM and was eventually replaced by Bacillus, which showed higher degree centrality in the HM network.

### 3.4 Biomarkers of the time series of breast milk microbial communities

**Figure 7:**
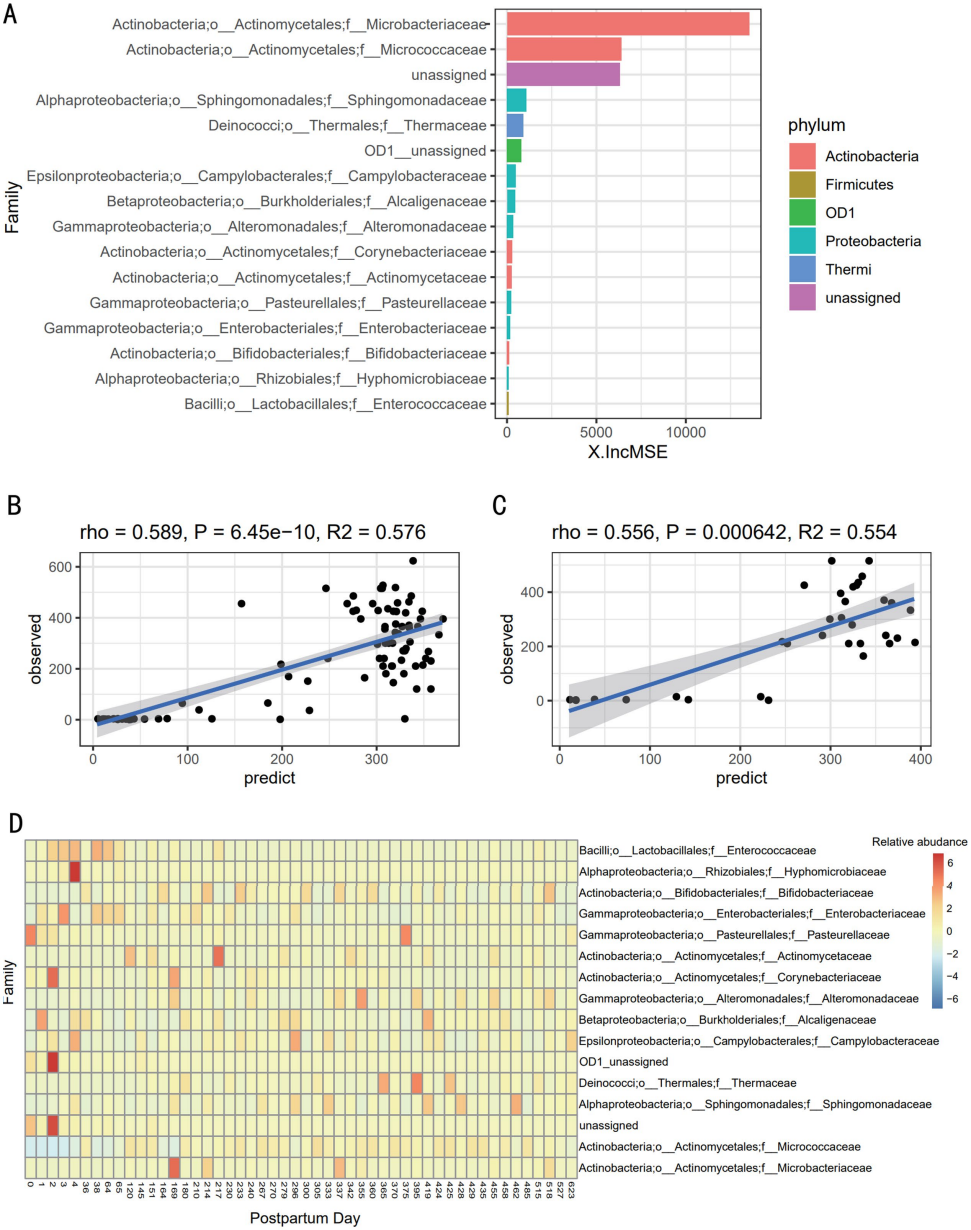
**(A)** Analysis results of forest regression model. **(B,C)** The variance of biomarkers in the training dataset and the validation dataset. **(D)** Heat map of microorganisms in HBM.

From the above results, we already know that there are significant differences in the structure and diversity of the microbial communities in HBM at each stage of lactation and that these differences are not affected by other factors. We used random forest and linear models to study HBM microbiota at the family level, genus level, and known species level, and those significantly correlated with DAY were used as biomarkers in the process of HBM maturation. We used randomly obtained samples (n = 150) as the training set for the random forest model, and used the remaining samples as the validation set. According to the random forest regression model results (Figure 7A), we found 16 families correlated with the time series namely, *Enterococcaceae, Hyphomicrobiaceae, Bifidobacteriaceae, Enterobacteriaceae, Pasteurellaceae, Actinomycetaceae, Corynebacteriaceae, Micrococcaceae, Alcaligenaceae, Alteromonadaceae, Micrococcacea, Campylobacteraceae, Sphingomonadaceae,*and *Thermaceae.*

However, because the microbial community structure in HBM samples at the same time point varied greatly among individuals, the explained variance (*R*^2^) of these biomarkers in the training data set was only 0.576 (Figure 7B), and the explained variance in the validation data set was 0.554 (Figure 7C).

From the heat map (Figure 7D), we can see that as DAY increased, the relative abundance of Pasteurellaceae decreased and that the relative abundances of *Enterococcaceae*, *Enterobacteriaceae*, and *Bifidobacteriaceae* showed a tendency to increase first and then decrease, while the relative abundances of aerobe including *Micrococcaceae*, *Sphingomonadaceae*, and *Thermalaceae* increased.

**Figure 8:**
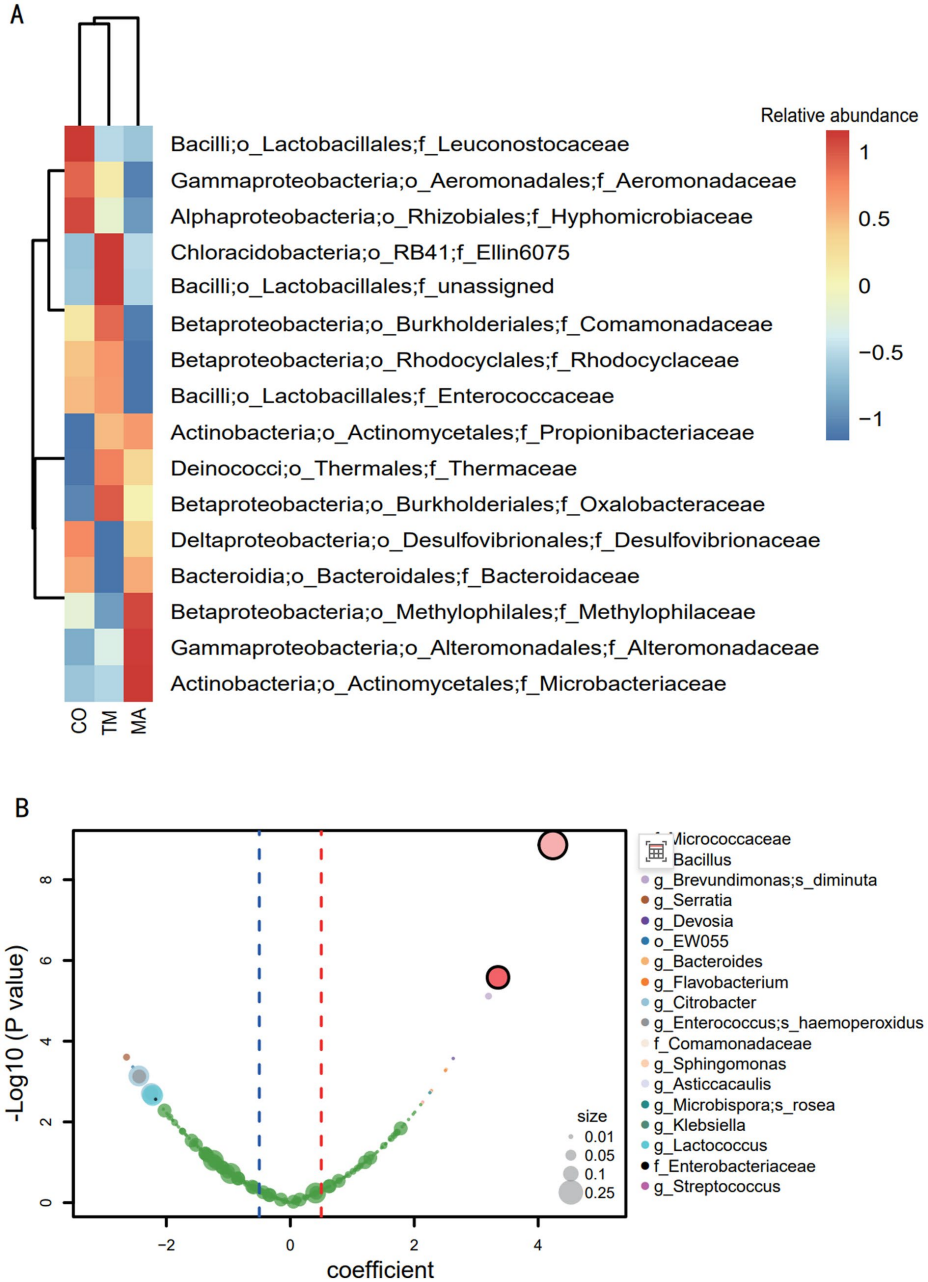
**(A)** Microbial families related to the lactation stage. **(B)** Distribution of association significance and coefficient of microbial taxa in the random forest model.

According to the random forest classification model, we also identified 16 families correlated with lactation stage (Figure 8A). The main biomarker families in HC were *Lactobacillaceae*, *Aeromonadaceae* and *Hyphomicrobiaceae*, and the main biomarker families in HM were *Methylophilaceae, Alteromonadaceae,* and *Microbacteriaceae*. The prediction accuracy of the classification model for breast feeding stage was 68.09%. Subsequently, we constructed a linear correlation model for DAY and the Pearson coefficient calculated according to the relative abundance of each microorganism at the genus level and the identifiable species level in HBM samples (Figure 8B).

We found core microorganisms that were positively correlated with DAY (FDR corrected *p* < 0.05, relative abundance. > 0.01%), including unassigned *Micrococcaceae, Bacillus, Streptococcus, Asticcacaulis, Sphingomonas, Flavobacterium*, and *Bacteroides* at the genus level and *Brevundimonas diminuta* and *Microbispora* at the species level; the core species that are negatively related include *Citrobacter, Lactococcus, Klebsiella, Serratia*, unassigned *Comamonadaceae*, and unassigned *Enterobacteriaceae* at the genus level and *Enterococcus haemoperoxidus* at the species level. The remarkable fact is that the correlation of relative abundance of some genera, due to the large differences between individuals, was not significantly with the days of lactation, but was significantly with the stage of lactation. For instance, the detection rate and average relative abundance of Bifidobacterium were significantly increased in TM (56.3, 0.037%) and HM (61.0, 0.092%) than HC (14.3, 0.007%).

## 4 Discussion

Research shows that HBM is an important source of the infant gut microbiota. By comparing the microbial composition of HBM and infant fecal samples, it was found that some bacterial species exist in both HBM and the infant gut, indicating that these microorganisms are likely transmitted from the mother to the infant through the breast feeding process. HBM is one of the key “seed” sources for the early colonization of gut microorganisms in infants (40, 41). Maternal human milk harbors its own microbiota consisting of numerous microbes; recent studies have started to vigorously explore this rich and diverse microbial community during breast feeding as a potential source for the microbial colonization in infant gut, which could substantially impact the establishment of infant gut microbiota (22, 42, 43). With the changes in the proportions of nutrients in HBM at different stages (44) and the oral microbes exchanged into HBM when babies suck (25, 45, 46), the structure and diversity of microbial communities in HBM also change with the passage of breast feeding time. Therefore, a comprehensive understanding of the temporal dynamics of the microbial community structure in HBM and the degree of influence of potential factors on HBM at different stages will help to understand the mechanisms underlying the origin of the HBM microbiome and the degree of controllability of infants obtaining microbes from HBM. And it is essential for understanding the maturation and healthy development of infant gut microbiota.

In this study, the 3 most dominant phyla were *Actinomycetota, Pseudomonadota* and *Bacillota*, and the most dominant families in these 3 phyla were *Micrococcaceae, Enterobacteriaceae, Streptococcaceae*, and *Staphylococcaceae*. However, there were large individual differences in the structure of the microbial community in HBM even at the phylum level alone. Among the tested variables, lactation stage was the primary determinant, explaining 18.5% of the variance in the HBM microbiome community structure and diversity. Given its dominant contribution relative to other factors, we defined reference ranges for the HBM microbiome across different lactation stages.

We performed dimensionality reduction and classification of the differences among HBM samples by hierarchical clustering based on bray curtis distance and obtained 4 clusters (C1-C4). We found that the distribution of HBM samples in each cluster had a significant temporal trend. From C1 to C4, the HBM samples corresponded to increasing DAY, indicating that among the factors tested in this study (delivery mode, IAP, maternal age, BMI, parity, and lactation stage), lactation stage was the strongest correlate of beta diversity in the HBM microbial community. It is important to note, however, that all measured factors together explained less than 20% of the total variance in the HBM microbiota (*R*^2^ = 0.194, *p* < 0.001), with lactation stage alone contributing 18.5% (*R*^2^ = 0.185, *p* < 0.001). Thus, the majority of the variability remains unexplained by the current set of variables. In addition, C1 and C2 were more similar with each other but had large differences among samples within each cluster; C3 and C4 were more similar with each other, with homogenous samples within each cluster. The main biomarker in C1 was *Staphylococcus*, that in C3 was *Sphingomonas*, and those in C4 were Unassigned *Enterobacteriaceae* and *Gluconobacter*. Although C2 had no unique biomarkers, the relative abundance of each biomarker in C2 was also significantly different from the other groups. And all these biomarkers may also be markers of beta diversity in HBM at different lactation stages.

In addition, we also found that mode of delivery has a significant effect on the alpha diversity in TM. The average alpha diversity in transitional HBM who underwent caesarean section was higher than that in transitional HBM who had a natural delivery, and the distribution within the group was relatively homogeneous. Regarding alpha diversity in HC microbiota, although there was no significant difference among the groups for different modes of delivery, in all HC samples from mothers in the caesarean section group, the alpha diversity showed substantial intragroup variations. These results may indicate that the mode of delivery mainly affects the microbial community in HC but that the degree of influence varies greatly among individuals. However, these individual differences were gradually reduced and disappeared with the maturation of HC.

Based on all the above results, the reason for this individual difference may be the IAP used by mothers who underwent caesarean section. It has been reported that *cephalosporins* and other commonly used IAP during caesarean section and postoperative recovery can enter the milk of lactating mothers (47, 48), which inevitably affects the community structure and diversity in the HBM microbiome. In addition, there are differences in the resistance to IAP among mothers (17, 41); that is, the endogenous route of the biological origin of the HBM microbiome is affected which also explains why the relative abundance of strictly anaerobic microorganisms in the HC from mothers who underwent caesarean section was much lower than that in other samples. As a result, IAP use leads to substantial individual differences in microbial community structure and alpha and beta diversity. With the increase in the DAY, the effects of IAP on HBM microbiota gradually dissipate due to pharmacokinetics; additionally, infants become more skilful at sucking, and the frequency of breast feeding increases. The close positive correlation between the ecological clusters composed of multiple microorganisms in the complex co-occurrence relationship network of HC microorganisms also adds evidence to this speculation regarding IAP. Different results relating to the effects of IAP during labour on HBM obtained from previous studies (17, 49). Different results relating to the effects of IAP during labour on HBM obtained from previous studies also seem to confirm this reasoning.

In addition to mode of delivery and IAP, we found that maternal age and parity also affect the microbial beta diversity in HBM. The main manifestation was that the relative abundance of *Staphylococcus, Enterococcus,* and *Anaerococcus* in the HBM from young primiparous mothers was slightly higher than that in the HBM from mothers in the other groups, and the relative abundance of unassigned *Microbacteriaceae* in the HBM of multiparous mothers was slightly higher. This may be because the gut microbiota of mothers of different ages varies (50, 51) or by the residual microbes in maternal mammary glands of the multiparous mothers that remained after breast feeding children previously. These microbes may have a great impact on HC. However, there are few reports on this in the current HBM research field, and a large number of HC samples are needed to further explore the influence of maternal age or gestational age on the structure of the HC microbial community. Nevertheless, mode of delivery, IAP, maternal age, parity and degree of obesity explained less than 1% of the structure and diversity of the microbial community in HBM and did not affect the intergroup differences in HBM microbial community structure and diversity at each stage of lactation. Among all the factors examined in this study, lactation stage explained the largest proportion of variance (*R*^2^ = 0.185, *p* < 0.001). However, it should be emphasized that all measured factors together accounted for less than 20% of the total variance, indicating that the majority of variability in the HBM microbiota remains unexplained by the current set of variables.

Some studies have shown that the microbial community of HBM consists of negatively correlated *Pseudomonadota* and *Bacillota* (25, 52). Moreover, in the HM of vaginal delivery group, we found that the relative abundance of Actinomycetota, was negatively correlated with that of *Pseudomonadota* and *Bacillota* but was positively correlated with the DAY, indicating that there are indeed microorganisms with a linear pattern with DAY. To verify this pattern, we used a random forest regression model and linear correlation model to find the 16 families that were most correlated with DAY. Through validation, we found that the accuracy of using these 16 families to predict the number of days of lactation was greater than 50% and that the accuracy of using these 16 families for predicting the breast feeding stage was nearly 70%. Except for *Microbacteriaceae* and *Alteromonadaceae*, the biomarkers determined by the 2 models were not the same. The different prediction accuracy rates and biomarkers also further illustrate the large individual differences among HBM samples, but the conventions we established can distinguish different HBM stages. The results also indicate that the differences in the abundance of *Microbacteriaceae* and *Alteromonadaceae* among HM samples are small and they are the most suitable biomarkers to determine the maturity of HBM.

And most of the bacteria, of which abundance increasing with maturation of HBM determined by random forests and correlation model are aerobic bacteria, such as skin common Micrococcaceae and environment common *Sphingomonadaceae* (*Sphingomonas*) and *Flavobacterium*. This result indicating that with increasing frequency and proficiency in breast feeding, there is a greater chance that aerobic bacteria from the mother’s skin and infant’s oral will flow back into the mammary gland with HBM. Breast feeding habits affect the microbial diversity of infants, and lower microbial diversity may be beneficial in the early stages of infants. It may help *Bifidobacterium* become the dominant flora, thereby promoting healthy gut colonization and immune development (25, 53), suggesting that the breast feeding can indeed significantly affect the HBM microbiome. Although the mothers who provided HBM in our study adopted the direct breast feeding (direct only) or mixed feeding (some indirect), it’s not just that mothers pass beneficial bacteria (such as *Lactobacillus* and *Bifidobacterium*) to their infants’ mouths through HBM. Bacteria in the infants’ mouths may also be transmitted back to the mothers’ mammary glands or HBM, thus affecting the microbial composition of the milk (46, 47), which emphasized the significant influence of infant breast feeding on the structure and diversity of the microbial community of HBM.

Among the core genera or species that have a significant correlation with DAY as determined by linear correlation models, the relative abundance of *Serratia* and *Klebsiella* (both belong to *Enterobacteriaceae*) was significantly negatively correlated with DAY, and *Serratia* and *Klebsiella* were the nodes with the highest degree in the correlation-based co-occurrence network of HC microorganisms. *Aeromonadaceae*, the biomarker in the HC stage determined by the random forest classification model, was among the nodes with the highest betweenness centrality in the correlation-based co-occurrence network of HC microorganisms. The core genus *Bacillus*, which has a significant positive correlation with DAY, is also the node with the highest degree and betweenness centrality in the HM correlation-based co-occurrence network. These observations suggest that the representative microorganisms at each lactation stage, such as *Serratia* in HC, *Klebsiella* and *Aeromonadaceae* in HM, also show high degree/betweenness centrality in the correlation network at their respective lactation stages.

Although we have found species that are potentially correlated with the maturity of HBM, we have not discovered the mechanism of action of these species in HBM at different stages of lactation. In the future, more samples will be needed, and the combination of transcriptome analysis and other methods will be used to further explore the mechanism of action of these microorganisms in HBM maturation, from the perspective of microbial functions and HBM nutrient structure.

### 4.1 Clinical implications

The clinical relevance of understanding temporal dynamics in breast milk microbiota is highlighted by several findings. For infants delivered by caesarean section, early and frequent breastfeeding can help restore beneficial bacterial transfer from the mother. The observation that IAP increases inter-individual variability in colostrum microbiota supports careful use of IAP and suggests consideration of probiotic supplementation for these mother-infant pairs. Stage-specific microbial biomarkers identified in this study may, in the future, aid in assessing breastfeeding adequacy or guiding interventions for dysbiosis. These implications require further validation through interventional studies.

## 5 Limitations

First, a limitation regarding the interpretation of delivery mode-related differences concerns confounding by IAP. As noted in the Methods, all mothers who underwent caesarean section received IAP according to standard clinical practice, whereas mothers with vaginal delivery did not. Therefore, observed differences in milk microbiota between delivery modes may be attributable to the mode of delivery itself, the use of IAP, or their interaction. Although we adjusted for IAP as a covariate in multivariate models, perfect collinearity with delivery mode precludes complete disentanglement. The temporal pattern (effects most pronounced in transitional milk and diminishing in mature milk) suggests that IAP alone does not explain all findings, but residual confounding cannot be fully excluded.

Second, this study did not collect information on infant sex or the number of siblings living in the same household. Have both factors been previously associated with early? life microbial exposure and could influence the composition of human milk microbiota, for example, through differential retrograde transfer from the infant’s oral cavity. The absence of these variables precludes adjustment for their potential confounding effects. Future studies should include infant sex and sibling number as covariates to more comprehensively assess determinants of the breast milk microbiome.

Third, detailed quantitative information on breastfeeding frequency, feeding intervals, and the intensity of sucking was not collected in this study. While we recorded that all participants exclusively breastfed during the first 5 months, we did not systematically document the number of feeds per day, the duration of each feed, or the proportion of exclusive versus mixed feeding over time. These behavioral variables can influence the degree of retrograde microbial transfer from the infant’s oral cavity to the mammary gland and may therefore affect the composition of the milk microbiota. As a result, breastfeeding behavior represents an important uncontrolled confounder in this study.

Fourth, this study relies on 16S rRNA V4-V5 amplicon sequencing, which has inherent technical limitations. Unlike shotgun metagenomics, 16S sequencing cannot resolve strains or provide species-level resolution for many genera due to high sequence similarity within the V4-V5 region. The choice of the V4-V5 primer set also introduces amplification bias, as primer binding efficiency varies across bacterial taxa, potentially leading to under- or over-representation of certain groups. The data are compositional in nature (relative abundances), which can introduce spurious correlations. Importantly, 16S sequencing does not provide direct information on microbial function, gene expression, or metabolic activity. Functional inferences presented in this study (e.g., aerobic/anaerobic phenotypes based on BugBase) are computational predictions and require validation by metatranscriptomics, metabolomics, or targeted culturing. Future studies employing shotgun metagenomics or multi-omics approaches are needed to overcome these limitations. Additionally, the co-occurrence networks are correlation-based and should be interpreted as exploratory statistical patterns, not as evidence of ecological interaction or causality.

Fifth, mature milk samples were collected at varying time points ranging from 16 to 856 days postpartum, which may introduce heterogeneity within the mature milk group. To mitigate this, we treated days of lactation as a continuous variable in regression analyses and used random forest regression to identify time-associated taxa. However, residual heterogeneity may remain, and future studies with more standardized collection timing (e.g., fixed days postpartum) are warranted.

Sixth, this study did not collect information on maternal education, occupation, household income, or detailed health status (e.g., chronic diseases, dietary intake, physical activity). These sociodemographic and health variables may influence the breast milk microbiome and could act as confounders or effect modifiers. The original questionnaire was designed to capture only the clinical and demographic factors most directly relevant to the research question (age, BMI, parity, delivery mode, IAP, feeding patterns, and lactation days). The absence of a full sociodemographic characteristics table is a limitation, and future studies should incorporate these variables to improve generalizability and reduce residual confounding.

## 6 Conclusion

In this study, we used a variety of statistical analysis methods and multiple models to explore the characteristics and potential influencing factors of HBM microbial community structure at different stages of lactation. The results show that compared with mode of delivery, IAP, degree of maternal obesity (BMI), age and parity and other factors on the structure and diversity of the HBM microbial community, Among the factors examined, lactation stage explained the largest proportion of variance (*R*^2^ = 0.185, *p* < 0.001), followed by delivery mode and IAP (each *R*^2^ < 0.01). All measured factors together explained less than 20% of the total variance, indicating that the majority of variability remains unexplained. Although individual HBM varies greatly, the significant differences in the structure and diversity of HBM microbial communities during each lactation stage can still be explained and distinguished by biomarkers at each stage. The mode of delivery and IAP during delivery only have a potential impact on HC microbes, implying the safety of HC from mothers who undergo caesarean section and emphasizing the importance of the initiation of breast feeding for infants delivered by caesarean section. All influencing factors in this study can only explain a small part of the structure and diversity of the microbial community in HBM, indicating that a large number of samples and multiple factors are still needed in the future for a more comprehensive understanding of the temporal dynamics and potential influencing factors of HBM microbiota.

## Data Availability

The 16S rRNA gene sequencing datasets used in this study are available in the National Center for Biotechnology Information (NCBI) Sequence Read Archive: http://www.ncbi.nlm.nih.gov/sra, accession number PRJNA663097.

## Footnotes

1
http://www.majorbio.com/

2
https://bugbase.cs.umn.edu/

3
http://huttenhower.sph.harvard.edu/galaxy/

## References

[1] Carr LE Virmani MD Rosa F Munblit D Matazel KS Elolimy AA . Role of human milk bioactives on infants’ gut and immune health. Front Immunol. (2021) 12:604080. doi: 10.3389/fimmu.2021.604080, 33643310 PMC7909314

[2] Masi AC Stewart CJ. Role of breastfeeding in disease prevention. Microb Biotechnol. (2024) 17:e14520. doi: 10.1111/1751-7915.14520, 38946112 PMC11214977

[3] Bardanzellu F Fanos V Reali A. “Omics” in human colostrum and mature milk: looking to old data with new eyes. Nutrients. (2017) 9:843. doi: 10.3390/nu9080843, 28783113 PMC5579636

[4] Sohn K Kalanetra KM Mills DA Underwood MA. Buccal administration of human colostrum: impact on the oral microbiota of premature infants. J Perinatol. (2016) 36:106–11. doi: 10.1038/jp.2015.157, 26658119

[5] Sakwinska O Moine D Delley M Combremont S Rezzonico E Descombes P . Microbiota in breast milk of Chinese lactating mothers. PLoS One. (2016) 11:e0160856. doi: 10.1371/journal.pone.0160856, 27529821 PMC4987007

[6] Meinzen-Derr J Poindexter B Wrage L Morrow AL Stoll B Donovan EF . Role of human milk in extremely low birth weight infants’ risk of necrotizing enterocolitis or death. J Perinatol. (2009) 29:57–62. doi: 10.1038/jp.2008.117, 18716628 PMC2801431

[7] Herrmann K Carroll K. An exclusively human milk diet reduces necrotizing enterocolitis. Breastfeed Med. (2014) 9:184–90. doi: 10.1089/bfm.2013.0121, 24588561 PMC4025624

[8] Tromp I Kiefte-de Jong J Raat H Jaddoe V Franco O Hofman A . Breastfeeding and the risk of respiratory tract infections after infancy: the generation R study. PLoS One. (2017) 12:e0172763. doi: 10.1371/journal.pone.0172763, 28231310 PMC5322970

[9] Zhang W Wang S Huang H Liu P Li J Deng Z . Color, macronutrients, fatty acids and sn-2 palmitoyl triacylglycerols (Opo/Opl) in human breast milk from Hainan Island of China: a comparison among different lactation periods. J Food Compos Anal. (2025) 148:108554. doi: 10.1016/j.jfca.2025.108554

[10] Roghair R. Breastfeeding: benefits to infant and mother. Nutrients. (2024) 16:3251. doi: 10.3390/Nu16193251, 39408217 PMC11478634

[11] Tomaszewska A Porębska K Jeleniewska A Królikowska K Lipińska-Opałka A Gościńska A . The home as a modulator of Milk immunity: association between domestic factors and immune cell populations in human breast Milk. Nutrients. (2025) 17:2574. doi: 10.3390/nu17152574, 40806158 PMC12348968

[12] Damaceno QS Souza JP Nicoli JR Paula RL Assis GB Figueiredo HC . Evaluation of potential probiotics isolated from human milk and colostrum. Probiotics Antimicrob Proteins. (2017) 9:371–9. doi: 10.1007/s12602-017-9270-1, 28374172

[13] Bzikowska-Jura A Czerwonogrodzka-Senczyna A Olędzka G Szostak-Węgierek D Weker H Wesołowska A. Maternal nutrition and body composition during breastfeeding: association with human milk composition. Nutrients. (2018) 10:1379. doi: 10.3390/nu10101379, 30262786 PMC6213543

[14] Perrin MT Fogleman AD Davis DD Wimer CH Vogel KG Palmquist AEL. A pilot study on nutrients, antimicrobial proteins, and bacteria in commerce-free models for exchanging expressed human milk in the Usa. Matern Child Nutr. (2018) 14:e12566. doi: 10.1111/mcn.12566, 30592165 PMC6866159

[15] Picaud JC Claris O Gil-Campos M De La Cueva IS Cornette L Alliet P . Partially hydrolyzed, whey-based infant formula with six human milk oligosaccharides, *B. infantis* LMG11588, and *B. lactis* CNCM I-3446 is safe, well tolerated, and improves gut health: a staged analysis of a randomized trial. Front Nutr. (2025) 12:2025.1628847/full. doi: 10.3389/fnut.2025.1628847, 40771223 PMC12325064

[16] Simpson MR Avershina E Storrø O Johnsen R Rudi K Øien T. Breastfeeding-associated microbiota in human milk following supplementation with *Lactobacillus rhamnosus* GG, *Lactobacillus acidophilus* La-5, and *Bifidobacterium animalis* ssp. lactis Bb-12. J Dairy Sci. (2018) 101:889–99. doi: 10.3168/jds.2017-13411, 29248229

[17] Zimmermann P Curtis N. Breast milk microbiota: a review of the factors that influence composition. J Infect. (2020) 81:17–47. doi: 10.1016/j.jinf.2020.01.023, 32035939

[18] Melekoglu E Yilmaz B Çevik A Yılmaz B Gökyıldız Sürücü Ş Avcıbay Vurgeç B . The impact of the human milk microbiota in the prevention of disease and infant health. Breastfeed Med. (2023) 18:413–30. doi: 10.1089/bfm.2022.0292, 37140562

[19] Ruiz L García-Carral C Rodriguez JM. Unfolding the human milk microbiome landscape in the omics era. Front Microbiol. (2019) 10:1378. doi: 10.3389/fmicb.2019.01378, 31293535 PMC6604669

[20] Meng L Xie H Li Z Tye KD Fan G Huang T . Gut-mammary pathway: breast milk microbiota as a mediator of maternal gut microbiota transfer to the infant gut. J Funct Foods. (2025) 124:106620. doi: 10.1016/j.jff.2024.106620

[21] Milani C Duranti S Bottacini F Casey E Turroni F Mahony J . The first microbial colonizers of the human gut: composition, activities, and health implications of the infant gut microbiota. Microbiol Mol Biol Rev. (2017) 81:e00036-17. doi: 10.1128/mmbr.00036-17, 29118049 PMC5706746

[22] Murphy K Curley D O’callaghan TF O’Shea C-A Dempsey EM O’Toole PW . The composition of human milk and infant faecal microbiota over the first three months of life: a pilot study. Sci Rep. (2017) 7:40597. doi: 10.1038/srep40597, 28094284 PMC5240090

[23] Chen H Yi B Qiao Y Peng K Zhang J Li J . Diversity-scaling analysis of human breast milk microbiomes from population perspective. Front Microbiol. (2022) 13:940412. doi: 10.3389/fmicb.2022.940412, 36225365 PMC9549050

[24] Li SW Watanabe K Hsu CC Chao SH Yang ZH Lin YJ . Bacterial composition and diversity in breast milk samples from mothers living in Taiwan and mainland China. Front Microbiol. (2017) 8:2017.00965/full. doi: 10.3389/fmicb.2017.00965, 28611760 PMC5447776

[25] Moossavi S Sepehri S Robertson B Bode L Goruk S Field CJ . Composition and variation of the human milk microbiota are influenced by maternal and early-life factors. Cell Host Microbe. (2019) 25:324–335.e4. doi: 10.1016/j.chom.2019.01.011, 30763539

[26] Gómez-Gallego C Morales JM Monleón D du Toit E Kumar H Linderborg KM . Human breast milk NMR metabolomic profile across specific geographical locations and its association with the milk microbiota. Nutrients. (2018) 10:1355. doi: 10.3390/nu10101355, 30248972 PMC6213536

[27] lackey KA Williams JE Meehan CL Zachek JA Benda ED Price WJ . What’s normal? Microbiomes in human milk and infant feces are related to each other but vary geographically: the inspire study. Front Nutr. (2019) 6:2019.00045/full. doi: 10.3389/fnut.2019.00045, 31058158 PMC6479015

[28] Liu S Mao Y Wang J Tian F Hill DR Xiong X . Lactational and geographical variation in the concentration of six oligosaccharides in Chinese breast milk: a multicenter study over 13 months postpartum. Front Nutr. (2023) 10:1267287. doi: 10.3389/fnut.2023.1267287, 37731395 PMC10508235

[29] Shama S Asbury MR Kiss A Bando N Butcher J Comelli EM . Mother’s milk microbiota is associated with the developing gut microbial consortia in very-low-birth-weight infants. Cell Rep Med. (2024) 5:101729. doi: 10.1016/j.xcrm.2024.101729, 39243753 PMC11525026

[30] Drago L Toscano M De Grandi R Grossi E Padovani EM Peroni DG. Microbiota network and mathematic microbe mutualism in colostrum and mature milk collected in two different geographic areas: Italy versus Burundi. ISME J. (2017) 11:875–84. doi: 10.1038/ismej.2016.183, 27983720 PMC5364364

[31] Ennis D Shmorak S Jantscher-Krenn E Yassour M. Longitudinal quantification of *Bifidobacterium longum* subsp. *infantis* reveals late colonization in the infant gut independent of maternal milk HMO composition. Nat Commun. (2024) 15:894. doi: 10.1038/s41467-024-45209-y, 38291346 PMC10827747

[32] khodayar-Pardo P Mira-Pascual L Collado MC Martínez-Costa C. Impact of lactation stage, gestational age and mode of delivery on breast milk microbiota. J Perinatol. (2014) 34:599–605. doi: 10.1038/jp.2014.47 24674981

[33] He S Chen C Cao L Nauta A Liu X Chen H . Metabolic diversity and competitive interactions of infant-derived bifidobacteria in human milk oligosaccharides and galacto-oligosaccharide utilization. J Dairy Sci. (2025) 108:9034–47. doi: 10.3168/jds.2025-26559, 40614808

[34] Alemán-Duarte MI Aguilar-Uscanga BR García-Robles G Ramírez-Salazar FDJ Benítez-García I Balcázar-López E . Improvement and validation of a genomic DNA extraction method for human breastmilk. Methods Protoc. (2023) 6:34. doi: 10.3390/mps6020034, 37104016 PMC10144544

[35] Xu R Gridneva Z Payne MS Nicol MP Cheema AS Geddes DT . Longitudinal profiling of the human milk microbiome from birth to 12 months reveals overall stability and selective taxa-level variation. Microorganisms. (2025) 13:1830. doi: 10.3390/microorganisms13081830, 40871334 PMC12388394

[36] Wu R Li D Zhang S Wang J Chen K Tuo Z . A pan-cancer analysis of the oncogenic and immunological roles of transglutaminase 1 (TGM1) in human cancer. J Cancer Res Clin Oncol. (2024) 150:123. doi: 10.1007/s00432-024-05640-6, 38472489 PMC10933153

[37] Augustine T Murugesan S Badri F Gentilcore G Grivel JC Akobeng A . Immunoglobulin-coating patterns reveal altered humoral responses to gut bacteria in pediatric cow milk allergies. J Transl Med. (2024) 22:1021. doi: 10.1186/s12967-024-05850-z, 39533360 PMC11558889

[38] Segata N Izard J Waldron L Gevers D Miropolsky L Garrett WS . Metagenomic biomarker discovery and explanation. Genome Biol. (2011) 12:R60. doi: 10.1186/gb-2011-12-6-r60, 21702898 PMC3218848

[39] Wang K Xia X Sun L Wang H Li Q Yang Z . Microbial diversity and correlation between breast Milk and the infant gut. Foods. (2023) 12:1740. doi: 10.3390/foods12091740, 37174279 PMC10178105

[40] Pärnänen K Karkman A Hultman J Lyra C Bengtsson-Palme J Larsson DGJ . Maternal gut and breast milk microbiota affect infant gut antibiotic resistome and mobile genetic elements. Nat Commun. (2018) 9:3891. doi: 10.1038/s41467-018-06393-w, 30250208 PMC6155145

[41] Xu D Wan F. Breastfeeding and infant gut microbiota: influence of bioactive components. Gut Microbes. (2024) 17:2446403. doi: 10.1080/19490976.2024.2446403, 39727058 PMC12931709

[42] Pannaraj PS Ly M Cerini C Saavedra M Aldrovandi GM Saboory AA . Shared and distinct features of human milk and infant stool viromes. Front Microbiol. (2018) 9:1162. doi: 10.3389/fmicb.2018.01162, 29910789 PMC5992295

[43] Dinleyici M Pérez-Brocal V Arslanoglu S Aydemir O Sevuk Ozumut S Tekin N . Composition of microbiota in transient and mature human milk: significant changes in large for gestational age group. Nutrients. (2024) 16:208. doi: 10.3390/nu16020208, 38257101 PMC10818272

[44] Biagi E Quercia S Aceti A Beghetti I Rampelli S Turroni S . The bacterial ecosystem of mother’s milk and infant’s mouth and gut. Front Microbiol. (2017) 8:1214. doi: 10.3389/fmicb.2017.01214, 28713343 PMC5491547

[45] Arishi RA Gridneva Z Perrella SL Cheema AS Lai CT Payne MS . Breastfeeding patterns and total volume of human milk consumed influence the development of the infant oral microbiome. J Oral Microbiol. (2025) 17:2469892. doi: 10.1080/20002297.2025.2469892, 40013012 PMC11864009

[46] Qi Q Wang L Zhu Y Li S Gebremedhin MA Wang B . Unraveling the microbial symphony: impact of antibiotics and probiotics on infant gut ecology and antibiotic resistance in the first six months of life. Antibiotics. (2024) 13:602. doi: 10.3390/antibiotics13070602, 39061284 PMC11274100

[47] Van Wattum JJ Leferink TM Wilffert B ter Horst PGJ. Antibiotics and lactation: an overview of relative infant doses and a systematic assessment of clinical studies. Basic Clin Pharmacol Toxicol. (2019) 124:5–17. doi: 10.1111/bcpt.13098 30015369

[48] Chen YY Zhao X Moeder W Tun HM Simons E Mandhane PJ . Impact of maternal intrapartum antibiotics, and caesarean section with and without labour on *Bifidobacterium* and other infant gut microbiota. Microorganisms. (2021) 9:1847. doi: 10.3390/microorganisms9091847, 34576741 PMC8467529

[49] Dong C Guan Q Xu W Zhang X Jin B Yu S . Disentangling the age-related manner in the associations between gut microbiome and women’s health: a multi-cohort microbiome study. Gut Microbes. (2023) 15:2290320. doi: 10.1080/19490976.2023.2290320, 38059752 PMC10730178

[50] huang S Haiminen N Carrieri AP Hu R Jiang L Parida L . Human skin, oral, and gut microbiomes predict chronological age. mSystems. (2020) 5:10.1128/msystems.00630-19. doi: 10.1128/msystems.00630-19, 32047061 PMC7018528

[51] Aslani S Mousavi SN Robati AK Heidarzadeh S Sabet SA. Composition in phyla from breast milk: effect of the mode of delivery. Rev Argent Microbiol. (2025) 57:231–8. doi: 10.1016/j.ram.2025.04.001, 40410009

[52] Yelverton CA Killeen SL Feehily C Moore RL Callaghan SL Geraghty AA . Maternal breastfeeding is associated with offspring microbiome diversity; a secondary analysis of the MicrobeMom randomized control trial. Front Microbiol. (2023) 14:1154114/full. doi: 10.3389/fmicb.2023.1154114, 37720155 PMC10502216

[53] Rodríguez JM. The origin of human milk Bacteria: is there a bacterial entero-mammary pathway during late pregnancy and lactation? Adv Nutr. (2014) 5:779–84. doi: 10.3945/an.114.007229, 25398740 PMC4224214

[54] Favara G Maugeri A Barchitta M Lanza E Magnano San Lio R Agodi A. Maternal lifestyle factors affecting breast milk composition and infant health: a systematic review. Nutrients. (2024) 17:62. doi: 10.3390/nu17010062, 39796495 PMC11723272

